# Identifying Conserved and Novel MicroRNAs in Developing Seeds of *Brassica napus* Using Deep Sequencing

**DOI:** 10.1371/journal.pone.0050663

**Published:** 2012-11-30

**Authors:** Ana Paula Körbes, Ronei Dorneles Machado, Frank Guzman, Mauricio Pereira Almerão, Luiz Felipe Valter de Oliveira, Guilherme Loss-Morais, Andreia Carina Turchetto-Zolet, Alexandro Cagliari, Felipe dos Santos Maraschin, Marcia Margis-Pinheiro, Rogerio Margis

**Affiliations:** 1 PPGGBM, Departamento de Genética, Universidade Federal do Rio Grande do Sul - UFRGS, Porto Alegre, Rio Grande do Sul, Brazil; 2 PPGBCM, Centro de Biotecnologia, Universidade Federal do Rio Grande do Sul - UFRGS, Porto Alegre, Rio Grande do Sul, Brazil; 3 Departamento de Botânica, Universidade Federal do Rio Grande do Sul - UFRGS, Porto Alegre, Rio Grande do Sul, Brazil; 4 Departamento de Biofísica, Universidade Federal do Rio Grande do Sul - UFRGS, Porto Alegre, Rio Grande do Sul, Brazil; Ecole Normale Supérieure de Lyon, France

## Abstract

MicroRNAs (miRNAs) are important post-transcriptional regulators of plant development and seed formation. In *Brassica napus*, an important edible oil crop, valuable lipids are synthesized and stored in specific seed tissues during embryogenesis. The miRNA transcriptome of *B. napus* is currently poorly characterized, especially at different seed developmental stages. This work aims to describe the miRNAome of developing seeds of *B. napus* by identifying plant-conserved and novel miRNAs and comparing miRNA abundance in mature versus developing seeds. Members of 59 miRNA families were detected through a computational analysis of a large number of reads obtained from deep sequencing two small RNA and two RNA-seq libraries of (i) pooled immature developing stages and (ii) mature *B. napus* seeds. Among these miRNA families, 17 families are currently known to exist in *B. napus*; additionally 29 families not reported in *B. napus* but conserved in other plant species were identified by alignment with known plant mature miRNAs. Assembled mRNA-seq contigs allowed for a search of putative new precursors and led to the identification of 13 novel miRNA families. Analysis of miRNA population between libraries reveals that several miRNAs and isomiRNAs have different abundance in developing stages compared to mature seeds. The predicted miRNA target genes encode a broad range of proteins related to seed development and energy storage. This work presents a comparative study of the miRNA transcriptome of mature and developing *B. napus* seeds and provides a basis for future research on individual miRNAs and their functions in embryogenesis, seed maturation and lipid accumulation in *B. napus*.

## Introduction

Eukaryotic gene expression is regulated at the transcriptional and post-transcriptional levels. An important post-transcriptional mechanism that has recently been discovered is controlled by endogenous, noncoding small RNAs (sRNAs), primarily small interfering RNAs (siRNAs) and microRNAs (miRNAs) [1]–[4]. In plants, miRNA genes, called primary miRNAs (pri-miRNAs), are typically encoded in intergenic regions and are transcribed by RNA Polymerase II as long polyadenylated transcripts, similar to protein-coding genes [5]. These primary sequences contain an imperfect stem-loop structure that is recognized by DICER-Like1 (DCL1) for sequential cleavage, which converts the pri-miRNAs into the precursor sequences (pre-miRNAs) that are further processed to generate 18–24 nucleotide (nt)-long sequences called mature miRNAs [6]. The imperfect complementary strand to the most abundant miRNA is often called miRNA*, and both strands are originated from the 5p and 3p arms of the pre-miRNA hairpin structure. These sRNAs play critical roles during plant development, regulating a variety of processes, such as embryogenesis, seed germination, organ formation, and developmental timing and patterning [7]–[13]. The binding of the miRNA to mRNA targets leads to gene silencing by endonucleolytic cleavage or translational inhibition, depending on the degree of complementarity between the miRNA and its target transcript [14]–[18].

*Brassica napus*, known as Oilseed Rape, is the third most important edible oil crop worldwide (www.faostat.fao.org). During embryogenesis, *B. napus* seeds build up storage reserves in specific tissues. The vast majority of these reserves are made up of lipids (40–45%) and proteins (17–26%) that are almost exclusively stored in the cotyledons of the maturing embryo [19]. Biogenesis of oil bodies (lipid-containing structures) begins as early as the heart stage in embryogenesis and lipid accumulation rapidly increases during weeks 4–8 after anthesis [20], [21]. These developmental stages are correlated with high synthetic lipid activity and a decline in the expression of genes coding for oil biosynthetic and glycolytic enzymes but not of the genes involved in the later steps of oil accumulation [22].

The number of miRNAs in the miRNA registry database (miRBase release 18) [23] that are known in *B. napus* (48 miRNAs) is considerably small when compared to the model plant *Arabidopsis* (328 miRNAs) or to the crop species soybean (395 miRNAs) and rice (661 miRNAs). *B. napus* (Bna) miRNAs were identified in a few previous studies, primarily through either a computational analysis of known plant miRNAs against the Bna Expressed Sequence Tag (EST) and Genome Survey (GSS) sequences [24] or cloning strategies from whole seedlings or vascular exudates of nutrient-stressed plants [25]–[29]. These strategies allowed for the identification of Bna-miRNAs that are conserved and highly abundant in many plant species [30], [31]. The application of high-throughput sequencing technology in functional studies of small RNAs has been useful in accelerating the discovery of low abundance and species-specific miRNAs in plants under several different growing conditions [32]–[40]. Recently, [41] nine new Bna-miRNAs were reported using deep sequencing to investigate the Bna-miRNA profiles of seeds from high and low oil-content cultivars in very early embryonic development stages. However, the expression patterns and functions of *B. napus* miRNAs from seed development stages to maturation remain largely unknown.

In the present work, we identified miRNAs that may be involved in stages of the *B. napus* seed development process and in the accumulation of storage reserves. Illumina sequencing of two small RNA libraries of immature and mature stages of *B. napus* seeds were used to characterize the miRNAs. In addition, polyadenylated transcript sequencing (mRNA-seq) libraries were used to identify the pre-miRNAs expressed in the seeds that were unknown to science. A total of 251 mature miRNAs from 59 distinct miRNA families were identified in the computational analysis, from which, 29 families were previously unidentified in *B. napus* but conserved in other plants and 13 families were reported for the first time in plants. Several miRNAs were more abundant in seed development stages than in mature seeds, and putative targets were predicted to encode a broad range of proteins related to seed development and energy storage.

## Materials and Methods

### Plant Material and Growth Conditions

*B. napus* (cultivar PFB-2, Embrapa) plants were grown in an open environment (30°S 51°W) from May to October 2010. Flowers were tagged upon opening and developing siliques from different plants were collected in the middle of a light cycle at 7, 14, 21, 28, 42 and 50 days after flower opening (DAF). The seeds were dissected from the siliques, immediately frozen in liquid nitrogen and stored at −80°C.

### RNA Isolation and Deep Sequencing

Total RNA was isolated from the seed material using Trizol (Invitrogen, CA, USA) according to the manufacturer’s protocol, and the RNA quality was evaluated by electrophoresis on a 1% agarose gel. Total RNA (>10 µg) was sent to Fasteris Life Sciences SA (Plan-les-Ouates, Switzerland) for processing. Two sRNA libraries were constructed; one from mature seeds (50 DAF) and one from an equivalent mixture of RNA from immature seeds at DAF stages 7–42. Briefly, the construction of the small RNA libraries consisted of the following successive steps: acrylamide gel purification of the RNA bands corresponding to the size range 20–30 nt, ligation of the 3p and 5p adapters to the RNA in two separate subsequent steps, each followed by acrylamide gel purification, cDNA synthesis followed by acrylamide gel purification, and a final step of PCR amplification to generate a cDNA colony template library for Illumina sequencing. The polyadenylated transcript sequencing (mRNA-seq) was performed using the following successive steps: Poly(A) purification, cDNA synthesis using Poly(T) primer, shotgun to generate inserts of 500 nt, 3p and 5p adapter ligations, pre-amplification, colony generation and Illumina single-end 100 bases sequencing. The libraries were sequenced by Illumina HiSeq2000.

### Data Analysis

The overall procedure for analyzing small libraries is shown in Figure S1. All low quality reads (FASTq value <13) were removed, and 5p and 3p adapter sequences were trimmed using Genome Analyzer Pipeline (Fasteris). The remaining low quality reads with ‘n’ were removed using PrinSeq script [42]. Sequences shorter than 18 nt and longer than 25 nt were excluded from further analysis. Small RNAs derived from Viridiplantae rRNAs, tRNAs, snRNAs and snoRNAs (from the tRNAdb [43], SILVA rRNA [44], and NONCODE v3.0 [45] databases] and small RNAs derived from Rosales mtRNA and cpRNA [from the NCBI GenBank database (http://ftp.ncbi.nlm.nih.gov)] were identified by mapping with Bowtie v 0.12.7 [46] and excluded from further miRNA predictions and analyses.

After cleaning the data (low quality reads, adapter sequences), the mRNA-seq data from the two libraries were pooled and assembled in contigs using the CLC Genome Workbench version 4.0.2 (CLC bio, Aarhus, Denmark) algorithm for *de novo* sequence assembly using the default parameters (similarity = 0.8, length fraction = 0.5, insertion/deletion cost = 3, mismatch cost = 3). In total, 237,993 contigs were assembled and used as reference for the discovery of pre-miRNA and miRNA sequences.

### Identification and Analysis of Conserved and Novel miRNAs

To identify plant-conserved miRNAs, small RNA sequences were aligned with known non-redundant plant mature miRNAs (Viridiplantae) and Brassicaceae precursors that were deposited in the miRBase database (Release 18, November 2011) using Bowtie v 0.12.7. Complete alignment of the sequences was required and zero mismatches were allowed. To search for novel miRNAs, small RNA sequences were matched against assembled mRNA-seq contigs using SOAP2 [47]. The SOAP2 output was filtered with an in-house filter tool to separate candidate sequences as miRNA precursors using an anchoring pattern of one or two blocks of aligned small RNAs with a perfect match. As miRNA precursors have a characteristic hairpin structure, the next step to select candidate sequences was secondary structure analysis by RNAfold using an annotation algorithm from the UEA sRNA toolkit [48]. In addition, perfect stem-loop structures should have the miRNA sequence at one arm of the stem and a respective antisense sequence at the opposite arm. Finally, precursor candidate sequences were checked using the BLASTn algorithm from the miRBase (www.miRBase.org) and NCBI databases.

For the frequency analysis of all identified miRNAs, sRNA reads were aligned in Bowtie v 0.12.7 using the default parameters, with the first seed alignment >28 nt in size and allowing zero mismatches. As reference, we used both previously annotated pre-miRNAs from miRBase and the putative pre-miRNAs identified in this work. The SAM files from Bowtie were then processed using Python scripts to assign the frequencies of each read and map them onto references. For data normalization, we use the scaling normalization method proposed by [49]. To assess whether the microRNA was differentially expressed, we independently used both the R package EdgeR [50] and the A–C test [51]. In brief, EdgeR uses a negative binomial model to estimate overdispersion from the miRNA count. The dispersion parameter of each miRNA was estimated by the tagwise dispersion. Then, differential expression is assessed for each miRNA using an adapted exact test for overdispersed data. The A-C test computes the probability that two independent counts of the same microRNA came from similar samples. We considered miRNAs to be differentially expressed if they had a p-value ≤0.001 in both statistical tests.

### Prediction of miRNA Targets

The prediction of target genes of novel miRNAs was performed against assembled RNA-seq contigs using psRNAtarget [52], with the default parameters and a maximum expectation value of 4 (number of mismatches allowed). Candidate RNA sequences were then annotated by assigning them putative gene descriptions based on their sequence similarity with previously identified and annotated genes that had been deposited in the NR and Swiss-Prot/Uniprot protein databases using BLASTx; this analysis was conducted using the blast2GO v2.3.5 software [53]. The annotation was improved by analyzing conserved domains/families using the InterProScan tool. Gene Ontology (GO) terms for the cellular component, molecular function and biological processes were determined using the GOslim tool in the blast2GO software. The orientation of the transcripts was obtained from BLAST annotations.

## Results

### Overview of B. napus RNAs Library Sequencing

**Figure 1:**
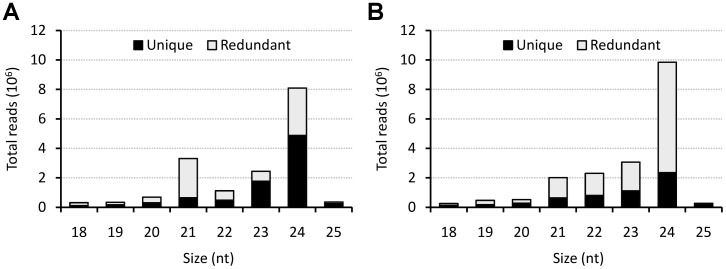
Length distribution and diversity of total number of small RNA reads of *B. napus* seed libraries. (**A**) Small RNA reads sequenced from the mature seed library. (**B**) Small RNA reads sequenced from the developing seed library.

To identify the miRNA transcriptome involved in *B. napus* seed development, sRNA libraries constructed from mature seeds and from an equivalent mix of immature seeds (a pool of DAF stages 7–42) were sequenced by using the Solexa/Illumina platform. Deep sequencing yielded a total of almost 38 million sRNA reads. After removing low-quality sequences, adapter contaminants and inserts, approximately 17 million and 19 million reads, with lengths ranging from 18 to 25 nt, were obtained from the mature and developing seed libraries, respectively (Table 1); these reads represented 8,632,807 and 5,665,721 of distinct sequences in each library, respectively (Table S1). Consistent with the length distribution pattern of sRNAs in other plant species, sequences between 21 to 24 nt long were the most abundant, with 24 nt long sRNAs as the main peak (Figure 1). A relatively large number of 22 and 23 nt long small RNAs were obtained in the developing seed dataset. This was previously observed in developing *B. napus* seed sRNA libraries [41]. The highest sequence redundancy was observed in the 21 nt long fraction of mature seed library and the 24 nt long fraction of the developing seed library (Figure 1 and Table S1). A small fraction from the total number of reads sequenced in the mature and developing seed libraries (10.2% and 2.2%, respectively) matched to miRNAs (Table 2). Approximately 4.3% and 2.9% of the reads matched non-coding sRNAs other than miRNAs (rRNA, tRNA, snRNA, snoRNA), respectively, and 3.7% and 0.5% matched organellar sRNAs (mtRNA, cpRNA), respectively. The majority of the reads did not match known small RNAs and possibly represent siRNAs.

**Table 1:** Summary of sequencing data of *B. napus* small RNA libraries.

	Mature seeds		Developing seeds	
Reads	Number of reads	Percentage (%)	Number of reads	Percentage (%)
Total reads*	17,878,538	100.0	19,954,089	100.0
18–25 nt	16,658,523	93.2	18,728,461	93.9
<18 nt	875,194	4.9	856,483	4.3
>25 nt	344,821	1.9	369,145	1.8

* High quality reads with lengths of 1 to 44 nt.

**Table 2:** Categorization of *B. napus* sequecences matching noncoding and organellar small RNAs.

	Mature seeds		Developing seeds	
sRNA*	Number of reads	Percentage (%)	Number of reads	Percentage (%)
miRNA	1,699,293	10.20	420,230	2.24
rRNA	675,151	4.05	524,132	2.80
tRNA	39,769	0.24	23,449	0.13
snRNA	2,688	0.02	1,830	0.01
snoRNA	1,911	0.01	1,567	0.01
mtRNA	298,127	1.79	44,370	0.24
cpRNA	316,543	1.90	51,188	0.27
other sRNA	13,625,041	81.79	17,661,695	94.30
Total	16,658,523	100	18,728,461	100

* Only 18–25 nt reads were considered. The small RNA were clustered according to their origin as follow: ribosome (rRNA); transporter (tRNA); small nuclear (snRNA); small nucleolar (snoRNA); mitochondrial (mtRNA) and chloroplastic (cpRNA).

Because the genome of *B. napus* is not publicly available, we sequenced the mRNA transcriptome of *B. napus* seeds for use as a reference sequence in further analysis. The pooled mRNA-seq yielded 32,485,023 reads, which were imported into the CLC Genomics Workbench and *de novo* assembled into 237,993 contigs with an average length of 284 bp. Contigs and non-assembled reads with a minimum length of 100 bp were further considered. The contigs ranged in size between the minimum set threshold of 100 bp and 12,344 bp (average size = 285 bp; N50 = 361 bp), with 29,157 contigs that were more than 500 bp in length.

### Identification of Conserved miRNAs in B. napus Seeds

**Figure 2:**
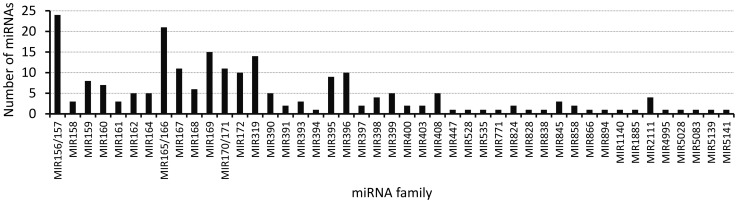
Number of miRNAs identified in *B. napus* seed libraries in known miRNA family in plants. The numbers are the sum of different miRNA containing the canonical sequences from the families of plant miRNAs deposited in miRBase.

There were 4,680 mature miRNAs from 52 Viridiplantae species deposited in the miRBase Release 18.0 from November 2011 [48]. Because miRNAs are highly conserved among plant species, the first approach to characterize the miRNA libraries was to precisely identify miRNAs by sequence homology. To identify conserved miRNAs in *B. napus* (Bna), the libraries were matched against the complete set of 2,585 unique plant mature miRNAs sequences from miRBase with no mismatches allowed. In total, 1,949,940 reads perfectly matched 219 known mature miRNA sequences, which corresponded to 45 plant miRNA families. On average, four miRNA members were identified within each miRNA family (Figure 2). Mature sequences matching MIR156 and MIR57, MIR165 and MIR166 or MIR170 and MIR171 were grouped as one single family due to their shared evolutionary origin. Of these reads, a total of 196 miRNAs were identified in the mature seed library, and 172 miRNAs were identified in the developing seed library, while 149 miRNAs were shared by both libraries (Table S2). From the total of 48 mature miRNAs annotated in miRBase for *B. napus* (Bna-miRNA), 24 unique Bna-miRNAs were detected in the libraries, representing all 17 known Bna-miRNA families. The remaining 28 miRNA families comprised miRNAs that are newly identified in *B. napus* but conserved in Brassicaceae species or among several plant species (Table 3 and Table S2). Overall, the largest family was MIR156/157, with 24 members representing MIR156/157 variants found in different species. MIR165/166 (21 members), MIR169 (15 members) and MIR319 (14 members) were the second, third and fourth largest miRNA families, respectively. Of the remaining miRNA families, 19 contained between 2 to 6 members, while 17 were represented by a single member.

**Table 3:** Number of miRNAs identified by sequence homology or matching pre-miRNAs in *B. napus* seed libraries that belong to novel and known plant miRNA families.

	Size									
Class	18	19	20	21	22	23	24	Total	Precursors	Families
New miRNAs known in other plants species (without precursor)^a^	0	7	40	122	17	0	2	**188**	0	**28**
Known miRNAs in B. napus (without precursor)^a^	0	0	0	21	3	0	0	**24**	0	**17**
New miRNAs in known *B. napus* families* (with precursor)^b^	0	1	7	7	1	0	0	**16**	21	**0 (11)** d
New miRNAs known in other plants species* (with precursor)^c^	0	0	1	6	1	0	0	**8**	8	**1 (6)** d
New miRNAs unknown in other plants species* (with precursor)^c^	1	0	0	12	2	0	0	**15**	15	**13**
**Total**	**1**	**8**	**49**	**171**	**24**	**0**	**2**	**251**	**44**	**59**

* most abundant;

a Data from Table S2;

b Data from Table S3;

c Data from Table S4;

d Number of identified families already considered in previous categories are in parenthesis.

### Identification of Novel B. napus miRNAs

To distinguish miRNAs from other small RNAs, such as siRNAs, some important features from miRNA biogenesis must be considered: 1) mature miRNAs are derived from pre-miRNAs; 2) all pre-miRNAs can form a secondary structure with a stem-looped hairpin; 3) the secondary structure shows high negative minimum folding free energy (MFE, 40–100 kcal/mol) and minimum folding free energy index (MFEI, higher than 0,85) [54]; 4) The stem-looped hairpin has the mature miRNA sitting on one of the arms and an almost complementary miRNA (with few mismatches) on their opposite site arm (5p and 3p positions). To identify novel miRNAs in *B. napus* seeds, the sRNA libraries were matched against assembled contigs of developing and mature seeds, because Bna ESTs and GSS were previously explored elsewhere [25], [24], [29], [41]. Candidate mRNA sequences with hairpin-like structures and with more than 10 miRNA reads that were anchored in the same orientation in the 5p and/or 3p arm in a two block-like pattern were considered putative pre-miRNAs. The MFE and MFEI were determined for each candidate sequence and the precursor identity was determined by BLAST searches against mature miRNAs at miRBase. As a result, three groups of pre-miRNAs were identified: (a) known in Bna, (b) new in Bna but known in plants and (c) new in Bna and uncharacterized in other plants.

The determined secondary structures of Bna pre-miRNAs identified in the first group showed an average MFE value of −57.16 kcal/mol, an average MFEI of −0.99 and an average GC content of 43.32% (Table S3). In addition, four mRNA sequences presented anchored miRNAs in a block-like pattern (Bna-MIR393-2; Bna-MIR393-2; Bna-MIR396; Bna-MIR1140) but did not fold into a secondary structure because they had partial mRNA sequences. However, these four sequences were considered an exception and were studied further because they showed high similarity to known Bna pre-miRNAs. In total, 17 new full-length and 4 partial pre-miRNA sequences were identified for 11 known Bna-miRNA families, along with 16 new 5p:3p pairs of mature Bna-miRNAs (Table 3 and Figure S2). It has been previously shown that miRNA variants, referred to as isomiRNAs, are detectable using high-throughput sequencing [36], [38], [55]. Known Bna-miRNAs and several novel isomiRNAs were detected in the predicted precursors (Table S3). The known Bna sequences represented the most abundant miRNA in eight of the 21 new precursors (Bna-MIR159, Bna-MIR166, Bna-MIR167, Bna-MIR168, Bna-MIR171 and Bna-MIR824) and were not considered new miRNAs in Table 3. The second group of new pre-miRNAs (Bna-nMIRx) comprised seven full-length and one partial pre-miRNA. Mature miRNA sequences of seven miRNA families (Bna-nMIR158, Bna-nMIR162, Bna-nMIR172, Bna-nMIR394, Bna-nMIR400, Bna-nMIR408 and Bna-nMIR827) that were not previously characterized in *B. napus* have been identified in these new pre-miRNAs (Table 3 and Table S4). The miRNA families MIR158 and MIR400 have been reported only in Brassicaceae species, whereas the other families are conserved in several plants. With the exception of one partial pre-miRNA (Bna-nMIR394), all of the new pre-miRNAs had 5p:3p arm miRNA pairs that were complementarily anchored (Figure S3). Several isomiRNAs were detected and are shown in Figure S3. Bna-nMIR827 showed one mismatch with other plant miRNAs and therefore it has not been detected on initial analysis (Table S2). The third group of pre-miRNAs comprised all sequences with characteristic hairpin-like structures with no homology to previously known plant miRNAs; these sequences were considered as novel pre-miRNAs in plants. To increase the reliability of the predictions, one additional criterion was considered: only candidate precursors with anchored mature sequences that could be found in both libraries or for which a complementary miRNA sequence could be identified in at least one library were annotated. As a result, 15 novel miRNAs, representing 13 novel Bna-miRNA families and distributed in 15 new precursors, were identified (Table 3). From these new miRNAs, 11 pre-miRNAs exhibited the 5p:3p miRNA pair (Figure S3). The average MFE value of the 23 newly predicted pre-miRNAs (plant conserved and novel) was −46.67 kcal/mol with a range of −8.7 to −131.5 kcal/mol (Table S4). The average length of the pre-miRNAs was 131 nt with a MFEI of −0.92 and a GC content of 39%. In accordance with previous results [33], the majority of the newly identified miRNA sequences had uracil (U) as their first nucleotide (Table S4).

### Expression Profile of B. napus miRNAs

**Figure 3:**
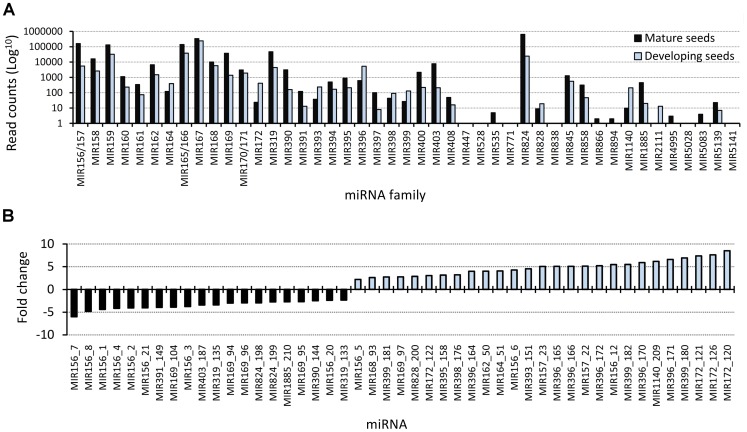
Sequencing profile of plant conserved miRNAs in *B. napus* seed libraries. (**A**) Number of total read counts of each miRNA in the mature and developing seed libraries of *B. napus*. (**B**) Mature miRNAs differentially expressed in the developing seed library and with fold-change higher than 2,0. Black bars represent miRNAs that were more abundant in mature seeds; blue bars represent miRNAs that were more abundant in developing seeds.

The large number of sequences produced by high-throughput sequencing enables the use of read counts in libraries as a reliable source for estimating the abundance of miRNAs [56], [57], [58]. The most abundant miRNAs identified by sequence homology in the libraries were MIR156, MIR159, MIR166, MIR167 and MIR824, each with more than 100,000 reads sequenced (Figure 3a). The majority of the conserved miRNAs that were identified had been sequenced less than 1,000 times, and 11 miRNA families had been detected less than 10 times. Although the total number of unique miRNAs detected in both libraries were similar, the number of total reads was higher in the mature seed library, where 1,581,402 reads (196 miRNAs) were identified, compared to 368,538 reads (172 miRNAs) in the developing seed library. A few poorly represented miRNA families, namely, MIR828 and MIR2111, were predominantly detected in the developing seed library (Figure 3a). Sharp differences in read abundance were also observed within members of one family and between miRNA libraries. For example, the abundance of MIR156/157 members ranged from 2 to 155 844 reads in the mature library and from 1 to 2 135 in the developing library. Comparisons between the normalized data suggested that 79 conserved miRNAs were differentially represented between the two libraries (Table S2). Differentially represented miRNAs in the developing seed library that exhibited more than a 2-fold change are shown in Figure 3b. Some members of MIR156/157, MIR162, MIR164, MIR168, MIR169, MIR172, MIR393, MIR395, MIR396, MIR398, MIR399, MIR828 and MIR1140 were more represented in developing seeds than in mature seeds (Figure 3b). On the contrary, some members of MIR156/157, MIR169, MIR319, MIR390, MIR391, MIR403, MIR824 and MIR1885 were more represented in mature seeds than in developing seeds (Figure 3b).

### Target Prediction of B. napus miRNAs

**Figure 4:**
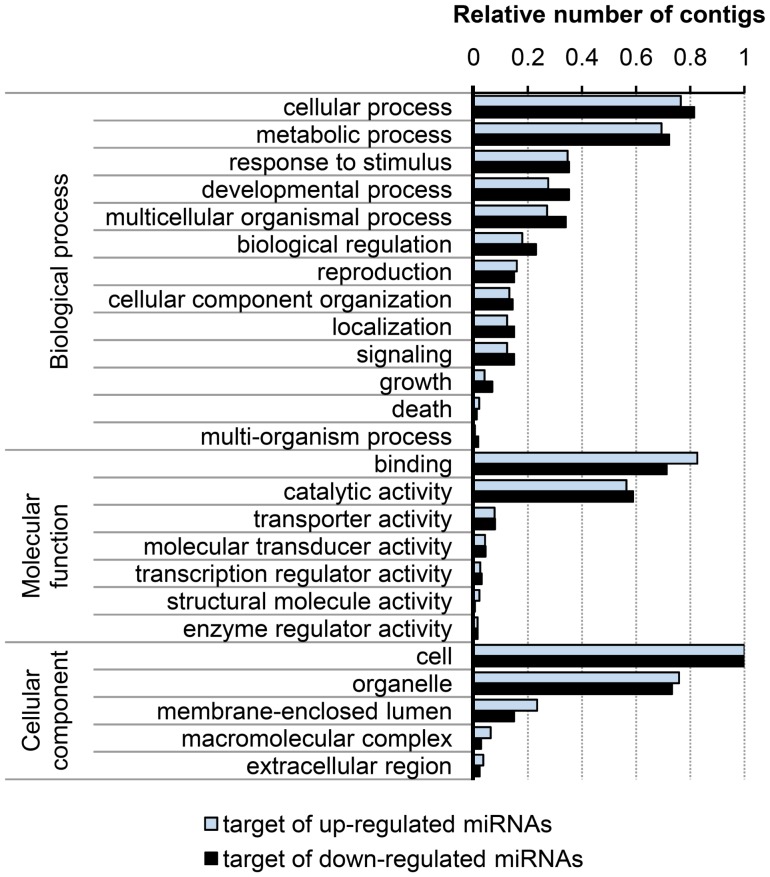
Targets of differentially expressed miRNAs in developing seeds of *B. napus.* The total number of contigs for each Gene Ontology (GO) term is relative to the total number of contigs in each gene category.

To infer the biological functions of the 23 newly identified miRNAs (plant conserved and novel), putative target genes were searched. The most abundant mature miRNAs were aligned to assembled *B. napus* contigs using the web-based computer server psRNATarget. Default parameters and a maximum expectation value of 4 (number of mismatches allowed) were used for higher prediction coverage. A total of 105 contigs matched miRNAs of the 14 novel and 8 known plant miRNA families identified in *B. napus*, representing 89 unique potential targets with an average of four targets per miRNA molecule. All of the identified targets were analyzed using a BLASTX against protein databases, followed by GO analysis to evaluate their putative functions. The detailed results of the best BLASTX hits are shown in Table S5. According to the categorization of GO annotation, 103 genes are involved in cellular components, with the majority of them localized in the nucleus and organelles. In the category of molecular functions, 103 genes participate in catalytic activities and binding activities with proteins and nucleic acids. With respect to biological processes, 95 genes primarily took part in responses to stimulus and different cellular and metabolic processes, suggesting that the novel Bna-miRNAs are involved in a broad range of physiological functions (Figure S4). We searched the putative target genes for differentially represented miRNAs and isomiRNAs shown in Table S2 and S3 to investigate whether these miRNAs regulate target genes involved in seed development and energy storage in *B. napus*. We found that 313 contigs were potential targets of 44 overrepresented miRNAs and 221 contigs were potential targets of 36 underrepresented miRNAs in the developing library (Table S6). In total, an average of seven targets per miRNA molecule was identified. According to the categorization of GO annotation, 436 genes are involved in cellular components, and 489 genes have been classified within categories of molecular function (Figure 4). With respect to biological processes (424 genes), miRNAs that were more abundant in mature than in developing seeds were found to potentially target genes that took part in growth, developmental processes, multicellular organismal process and biological regulation, along with different cellular and metabolic processes and responses to stimulus (Figure 4).

## Discussion

MiRNAs have been shown to play critical roles in the regulation of gene expression during plant development and in species-specific adaptation processes. In this study, we profiled by deep sequencing the microRNAome of the mature and immature stages of *B. napus* seeds. A total of 59 miRNA families were detected in the sRNA libraries (Tables S2 and 3). The families detected here increase the number of 17 miRNA families previously described in *B. napus* in the miRBase registry. We describe 29 miRNA new families in *B. napus* but conserved in other plants, and 13 families that were reported for the first time in plants.

A large number of reads were sequenced from both miRNA libraries of developing seeds and mature seeds of *B. napus*, providing a good representation of the miRNA population in seeds (Table 1). As expected, most of the highly conserved miRNAs in diverse plant species were also the most abundant in *B. napus* seeds. In addition, the conserved miRNA families of *B. napus* showed the higher number of members per family [30], [31]. miRNA families described only in Brassicaceae species were identified (MIR158, MIR161, MIR391, MIR400, MIR447, MIR771, MIR824, MIR838, MIR858 and MIR866) along with two families (MIR1885 and MIR1140) that could be specific to the genus *Brassica*. MIR1885, which was previously only identified in *B. rapa*, has been detected in the present *B. napus* libraries, and Bna MIR1140 was recently detected in *B. rapa* [39]. These results suggest that both the ancient regulatory pathways mediated by evolutionarily conserved miRNAs as well as novel and specialized pathways unique to Brassicaceae species, are present in *B. napus* [30], [31]. Nearly all of the unique Bna-miRNA sequences described in miRBase were detected, and all of the Bna-miRNA families were represented in at least one library. In addition, the identification of Bna pre-miRNAs allowed for the identification of several isomiRNAs that were identical to some conserved miRNAs identified in Table S2 and often more abundant than the known Bna sequences (Tables S2 and S3). Because the Bna-miRNA sequences deposited in miRBase were mainly identified by sequence homology and cloning of miRNAs isolated from whole plant tissues, it is tempting to conclude that the most abundant miRNA sequences detected in this study are seed specific. Although similar conclusions were proposed in rice [34], this observation is likely to reflect the increased detection power of the deep sequencing strategy and the limited computational analysis due to an incomplete genome.

To predict new miRNAs with confidence, we identified precursor sequences according to the strict criteria of having sharply defined distribution patterns of one or two block-like anchored sRNAs and at least 11 reads in total. It was previously found that the read depth distribution along putative pre-miRNAs can be used as a reliable guide for differentiating possible miRNAs from contaminant sequences, such as degradation products of mRNAs or transcripts that are simultaneously expressed in both the sense and antisense orientations [59]. In addition, the average MFE and MFEI of the predicted stem-loop structures of the pre-miRNAs values were within the confidence values suggested by [54] and are similar to the length average, MFE, MFEI and GC content of pre-miRNAs from other plant species, such as Arabidopsis [60]. For the majority of the new miRNAs, the complementary miRNA species (5p:3p pairs) were detected in our libraries, providing strong evidence that they derived from precisely processed stem-loops during miRNA biogenesis [6]. During the preparation of this manuscript, [41] reported nine new miRNA families in the very early stages of *B. napus* seed development. Bna-nMIR03, which was detected in both the developing and mature libraries in the present study, showed an identical sequence to one of the miRNA families presented by [41]. Taken together, these results strongly suggest that genuinely new Bna-miRNAs have been predicted, and also demonstrate that using a combination of sRNA and mRNA sequencing is a powerful strategy to discover new miRNAs in plants without an available genome.

In this study, 23 new miRNAs have been identified in *B. napus* seed libraries (Table S4). Furthermore, several miRNAs and isomiRNAs were more represented in developing seeds and may regulate the expression of target genes involved in seed development and energy storage in *B. napus* (Figure 3b, Tables S2 and S3). To infer about the biological significance of the results, *in silico* target predictions with the permissive expectation value of 4 were chosen. This strategy, which can include false targets, were previously used to successful predict true alternative targets that can be species or tissue-specific [61], [62]. GO annotation analyses suggested that miRNAs more abundantly present in mature seeds are probably involved in the down-regulation of genes that are more important during seed development, namely genes related to auxin signaling (ARFs, F-boxes, auxin efflux carrier component) or essential transcription factors in the regulation of plant development (NAC, SCL, TOE) [63]–[65]. Because the accumulation of dry matter for seed germination is the main priority of developing seeds, a large number of target genes may participate in these processes. Interestingly, some of the targets from the differentially abundant MIR156, MIR167, MIR169, MIR171, MIR319 and MIR396 were related to lipid metabolism (Table S6). Defects in ethanolamine-phosphate cytidylyltransferase, which is the target of Bna-nMIR04 and is predicted to be involved in lipid metabolic process, have been shown to affect embryonic and postembryonic development in Arabidopsis [66]. In conclusion, some potentially valuable targets emerge from the analysis that would be interesting to validate. Further investigation in the role of seed-specific miRNAs will contribute to the knowledge of the energy storage process in seeds.

This work provides a comparative study of the miRNA content of the transcriptome of mature and developing seeds of *B. napus*. The results will support future research on deeper studies of individual Bna-miRNAs and their function on embryogenesis and seed maturation. It is clear that the identification of miRNAs is not yet complete in *B. napus* and that the release of the genome sequences will be essential to fully understand the complete miRNA repertoire. One future endeavor is to look for more novel miRNAs; however, expression analysis and target validation will be critical for determining the biological functions of both the conserved and novel miRNAs identified during each developing seed stage of different *B. napus* cultivars.

### Accession Numbers

Sequencing data is available at the NCBI Gene Expression Omnibus (GEO) ([http://www.ncbi.nlm.nih.gov/geo]). The accession number GSE38020 contain the sequence data of mature and developing seed libraries from mRNA-seq and sRNA-seq.

## Supporting Information

Figure S1 **Flow chart of the procedure for the identification of miRNAs.**
(TIF)

Figure S2 **Predicted secondary structures of the new pre-miRNAs of known** ***B. napus*** **miRNA families.** Secondary structures and the locations of the miRNAs mapped onto these precursors. Mature miRNAs located in the 5p and 3p arms are labeled in magenta and red, respectively.
(PDF)

Figure S3 **Predicted secondary structures of new pre-miRNAs in** ***B. napus*** **.** Secondary structures of candidate miRNA precursors of new *B. napus* miRNA families (Bna-nMIRx), their locations and the abundance of small RNAs mapped onto these precursors. Mature miRNAs located in the 5p and 3p arms are labeled in magenta and red, respectively. Values on the left side of the miRNA sequence represent read counts in the mature and developing seed libraries, respectively.
(PDF)

Figure S4 **Distribution of target genes of new Bna-miRNAs in gene categories and Gene Ontology (GO) terms.**
(TIF)

Table S1 **Length distribution of raw reads of** ***B. napus*** **small RNAs libraries.**
(XLS)

Table S2 **New and Bna-known miRNAs identified in small RNA libraries.** *B. napus* miRNAs with perfect homology to plant miRNAs deposited in the miRBase database (release 18, November 2011).
(XLS)

Table S3 **New and Bna-known putative miRNA precursors identified in** ***B. napus*** **miRNA families.**
(XLS)

Table S4 **New miRNAs and pre-miRNAs identified in** ***B. napus*** **libraries.** Sequences were classified as known or as novel plant miRNA families.
(XLS)

Table S5 **Predicted targets of novel and plant conserved miRNAs in** ***B. napus*** **.**
(XLS)

Table S6 **Predicted targets of differentially expressed miRNAs in** ***B. napus*** **.**
(XLS)
