# Supplementary figures and images for: Identifying Conserved and Novel MicroRNAs in Developing Seeds of Brassica napus Using Deep Sequencing
Source: PLoS One. 2012 Nov 30;7(11):e50663. doi: 10.1371/journal.pone.0050663 (PMC3511302; doi:10.1371/journal.pone.0050663)

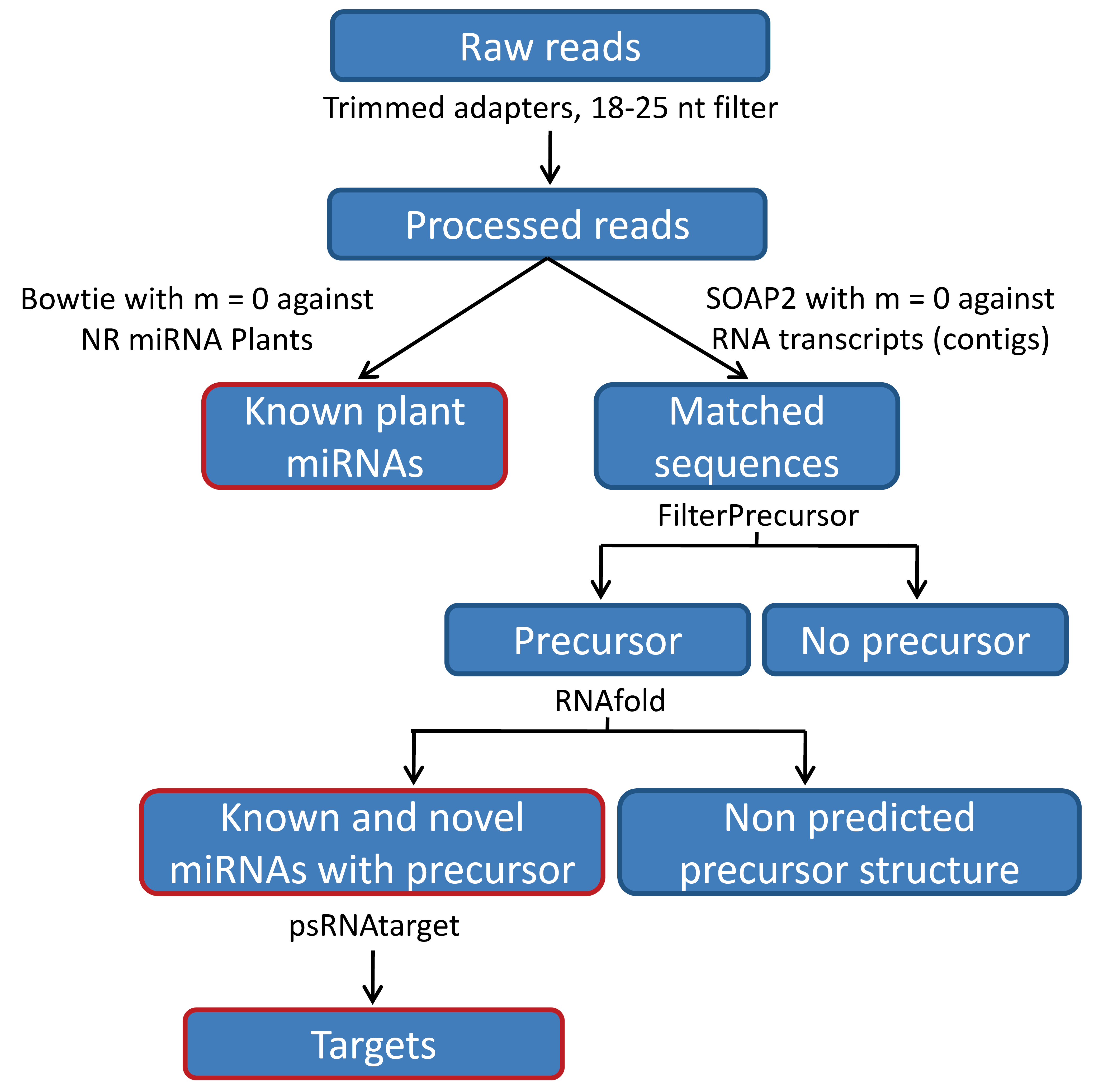

Supplement: Figure S1 — Flow chart of the procedure for the identification of miRNAs. (TIF) [file pone.0050663.s001.tif]

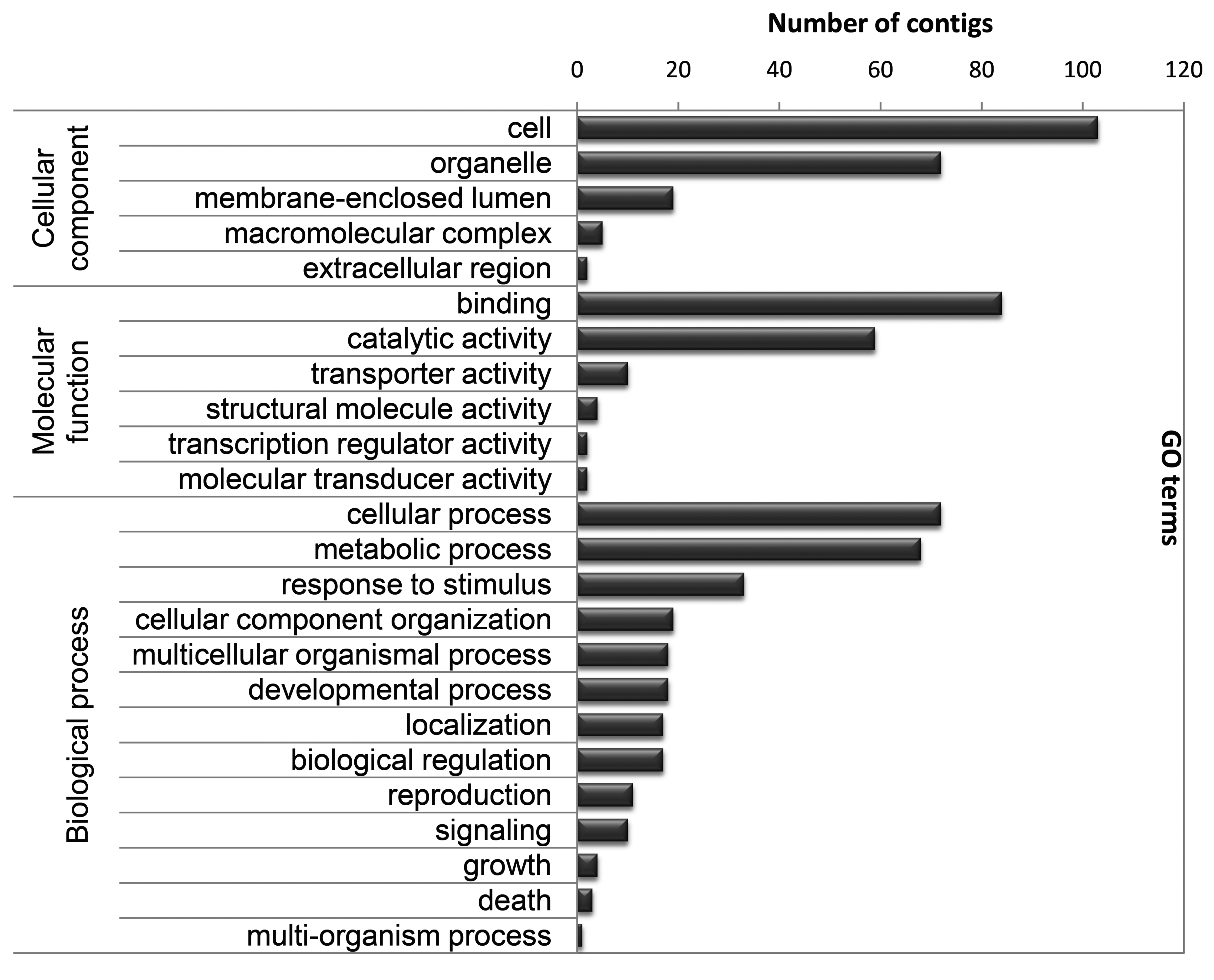

Supplement: Figure S4 — Distribution of target genes of new Bna-miRNAs in gene categories and Gene Ontology (GO) terms. (TIF) [file pone.0050663.s004.tif]
