# Supplementary material for: Identifying Conserved and Novel MicroRNAs in Developing Seeds of Brassica napus Using Deep Sequencing
Source: PLoS One. 2012 Nov 30;7(11):e50663. doi: 10.1371/journal.pone.0050663 (PMC3511302; doi:10.1371/journal.pone.0050663)

Fig. S2. Secondary structures of the new *B.napus* miRNA candidate precursors of known plant miRNA families and location small RNAs mapped to these precursors.

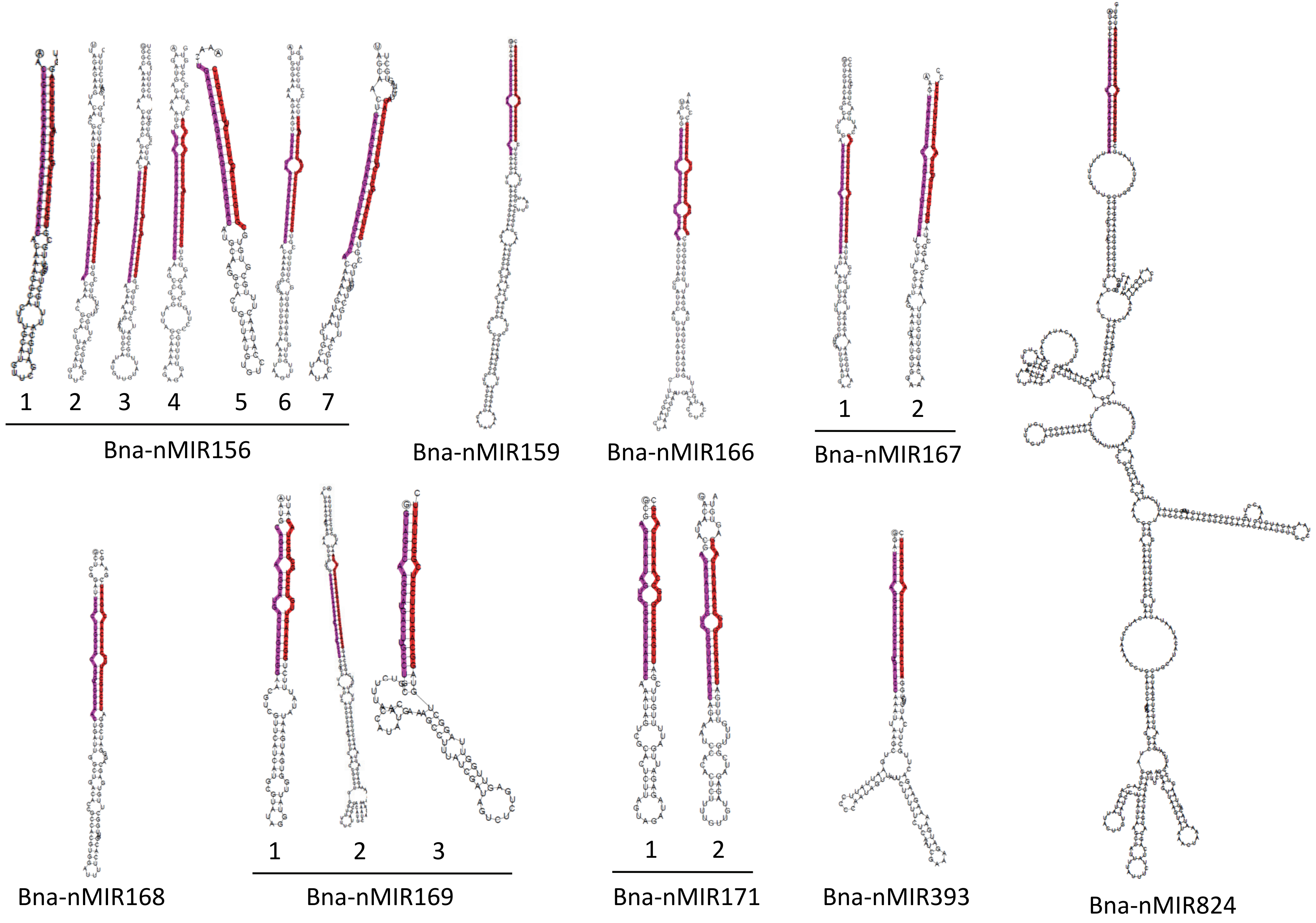

Supplement: Figure S2 — Predicted secondary structures of the new pre-miRNAs of known B. napus miRNA families. Secondary structures and the locations of the miRNAs mapped onto these precursors. Mature miRNAs located in the 5p and 3p arms are labeled in magenta and red, respectively. (PDF) [file pone.0050663.s002.pdf]
