# Supplementary material for: Identifying Conserved and Novel MicroRNAs in Developing Seeds of Brassica napus Using Deep Sequencing
Source: PLoS One. 2012 Nov 30;7(11):e50663. doi: 10.1371/journal.pone.0050663 (PMC3511302; doi:10.1371/journal.pone.0050663)

Fig. S3. Predicted secondary structures of new miRNA candidates in the *B. napus*. Secondary structures of candidate miRNA precursors of new *B. napus* miRNA families (Bna-nMIRx), their locations and the expression of small RNAs mapped onto these precursors. Sequences of miRNA candidates located in the 5p and 3p arms are labeled in purple and red, respectively. Values on the left side of the miRNA sequence represent read counts in the mature and developing seed libraries, respectively.

Bna-nMIR158

```
                        1 [         .         .         .         .         :         .         .         .         . ] 92
mir158        100.0%  GTTATCTCTGTTCTTCTTTGTCTATCGTTTGGAAAAGATTGATGACGCCATCTACACTTTTCCAAATGTAGACAAAGCAAAACCGTGATGAC
4570_19743    100.0%  ---------------CTTTGTCTATCGTTTGGAAAAG-------------------------------------------------------
1841_525      100.0%  -----------TCTTCTTTGTCTATCGTTTGGA-----------------------------------------------------------
146_388       100.0%  ---------------CTTTGTCTATCGTTTGGAAAAGA------------------------------------------------------
301_56        100.0%  ----------------TTTGTCTATCGTTTGGAAAAGA------------------------------------------------------
95_156        100.0%  ---------------CTTTGTCTATCGTTTGGAAAA--------------------------------------------------------
56_119        100.0%  ----------------TTTGTCTATCGTTTGGAAAAG-------------------------------------------------------
17_41         100.0%  ---------------CTTTGTCTATCGTTTGGAAA---------------------------------------------------------
9_41          100.0%  ---------------CTTTGTCTATCGTTTGGA-----------------------------------------------------------
128_33        100.0%  -----------TCTTCTTTGTCTATCGTTTGGAA----------------------------------------------------------
0_20          100.0%  ---------------CTTTGTCTATCGTTTGGAAAAGAT-----------------------------------------------------
7_13          100.0%  ---------------CTTTGTCTATCGTTTGGAA----------------------------------------------------------
2_10          100.0%  ------------------TGTCTATCGTTTGGAAAAG-------------------------------------------------------
0_8           100.0%  ---------------CTTTGTCTATCGTTTGGAAAAGATT----------------------------------------------------
14_3          100.0%  -----------TCTTCTTTGTCTATCGTTTGG------------------------------------------------------------
6_1           100.0%  ------------CTTCTTTGTCTATCGTTTGGAA----------------------------------------------------------
51040_3533    100.0%  ----------------------------------------------------------TTTCCAAATGTAGACAAAGCA-------------
3557_361      100.0%  -----------------------------------------------------------TTCCAAATGTAGACAAAGCA-------------
2542_149      100.0%  ----------------------------------------------------------TTTCCAAATGTAGACAAAGC--------------
633_32        100.0%  ----------------------------------------------------------TTTCCAAATGTAGACAAAG---------------
160_12        100.0%  ----------------------------------------------------------TTTCCAAATGTAGACAAA----------------
107_13        100.0%  ---------------------------------------------------------TTTTCCAAATGTAGACAAAGC--------------
68_6          100.0%  ----------------------------------------------------------TTTCCAAATGTAGACAAAGCAA------------
60_18         100.0%  -------------------------------------------------------------CCAAATGTAGACAAAGCA-------------
57_2          100.0%  ------------------------------------------------------------TCCAAATGTAGACAAAGCA-------------
46_3          100.0%  -----------------------------------------------------------TTCCAAATGTAGACAAAGC--------------
12_1          100.0%  ---------------------------------------------------------TTTTCCAAATGTAGACAAAGCA-------------
9_7           100.0%  -------------------------------------------------------------CCAAATGTAGACAAAGCAAAAC---------
8_2           100.0%  -----------------------------------------------------------TTCCAAATGTAGACAAAG---------------
3_23          100.0%  --------------------------------------------------------------CAAATGTAGACAAAGCAAAAC---------
```

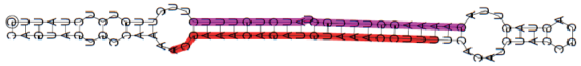

Bna-nMIR162

```
                         1 [         .         .         .         .         :         .         .         .         .         1         .    ] 116
mir162      100.0%   AGGAGTAAAAGATGCTGGAGGCAGCGGTTGATCGATCTCTTTGTGGGAATTTTTGAAAAACAAAATTAAAAGCATGAACAGATCGATAAACCTCTGCATCCAGCGGTCGCCTCTTC
29_21       100.0%   ----------------GGAGGCAGCGGTTGATCGATC-------------------------------------------------------------------------------
6790_1470   100.0%   ----------------------------------------------------------------------------------TCGATAAACCTCTGCATCCAG-------------
17_5        100.0%   ------------------------------------------------------------------------------------GATAAACCTCTGCATCCAG-------------
16_5        100.0%   ----------------------------------------------------------------------------------TCGATAAACCTCTGCATC----------------
16_4        100.0%   ----------------------------------------------------------------------------------TCGATAAACCTCTGCATCCAGC------------
14_4        100.0%   -----------------------------------------------------------------------------------CGATAAACCTCTGCATCCAG-------------
8_2         100.0%   ----------------------------------------------------------------------------------TCGATAAACCTCTGCATCCA--------------
5_1         100.0%   ----------------------------------------------------------------------------------TCGATAAACCTCTGCATCC---------------
```

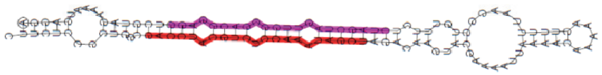

Bna-nMIR172

```
                         1 [         .         .         .         .         :         .         .         .         .         1  ] 103
mir172      100.0%   TGCTATTGCATCATCTTCAAGATTCAGAAATCATATTCTTTGATGGGTTCTCCTCTGATCCCATCTTCTTTGGTTTGAGAATCTTGATGATGCTGCAGCGGCA
16_1424     100.0%   -------GCATCATCTTCAAGATTCAGA---------------------------------------------------------------------------
2_209       100.0%   -----------------------------------------------------------------------------AGAATCTTGATGATGCTGCAG-----
0_6         100.0%   ----------------------------------------------------------------------------GAGAATCTTGATGATGCTGCA------
```

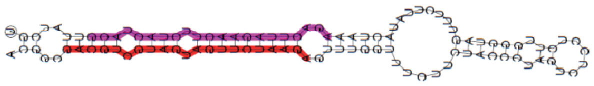

Bna-nMIR400

```
                         1 [         .         .         .         .         :         .         .         .         .         1         .         . ] 122
mir400-1     100.0%   AGAAGTTGCTGAAGATTGTTTTATGAGAGTATTATAAGTCACCGCATTTGGTAATCAAGTGTTGCTTTCCCAAACGAAGTGACTTATAAAGATCTCATGATTAATCGATTTTTTGCAAGGTC
2173_219     100.0%   ---------------------TATGAGAGTATTATAAGTCAC--------------------------------------------------------------------------------
240_14       100.0%   ---------------------TATGAGAGTATTATAAGTCA---------------------------------------------------------------------------------
15_3         100.0%   --------------------TTATGAGAGTATTATAAGTCA---------------------------------------------------------------------------------
74_5         100.0%   ---------------------TATGAGAGTATTATAAGT-----------------------------------------------------------------------------------
35_3         100.0%   ---------------------TATGAGAGTATTATAAGTC----------------------------------------------------------------------------------
5_1          100.0%   ----------------------ATGAGAGTATTATAAGTCAC--------------------------------------------------------------------------------
3_1          100.0%   --------------------------------------------------------------------------------GACTTATAAAGATCTCATGAT---------------------
```

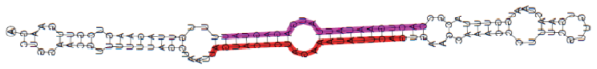

```
                         1 [         .         .         .         .         :         .         .         . ] 86
mir400-2     100.0%   TGTTTATGAGAGTATTATAAAGTCACTACGTTTGGTAATCAACTGTTGCTTTCCGTAACGAAGTGACTTATAATAATCTCATGAAT
130_19       100.0%   ----TATGAGAGTATTATAAAGTCAC------------------------------------------------------------
24_1         100.0%   ----TATGAGAGTATTATAAAGTCA-------------------------------------------------------------
7_0          100.0%   ----TATGAGAGTATTATAAAGT---------------------------------------------------------------
6_1          100.0%   ----------------------------------------------------------------GACTTATAATAATCTCATGA--
3_2          100.0%   ----------------------------------------------------------------GACTTATAATAATCTCATGAA-
```

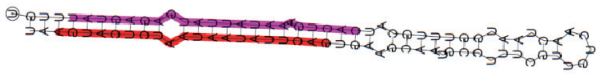

Bna-nMIR408

```
                 1 [         .         .         .         .         :         .         .         .         .         1         .         .         .         .       ] 149
mir408   100.0%  TAAAGAAGACAAAAGTGGTAACCAGAGAGAGACAGGGAACAAGCAGAGCATGGATTGAGTTTTTACCAAAACATCAAATGACTATGTTTGGTCTCTTCCCATGCACTGCCTCTTCCCTGGCTCCCTGTTTCTCCTCCCTTCTCTTTTTT
87_70    100.0%  -------------------------------ACAGGGAACAAGCAGAGCATG-------------------------------------------------------------------------------------------------
25_5     100.0%  -----------------------------------------------------------------------------------------------------TGCACTGCCTCTTCCCTGGCT---------------------------
20_11    100.0%  ----------------------------------------------------------------------------------------------------ATGCACTGCCTCTTCCCTGGC----------------------------
```

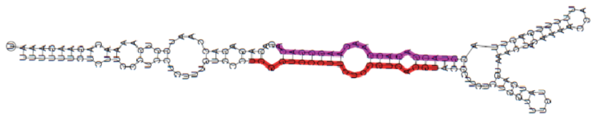

Bna-nMIR827

```
                    1 [         .         .         .         .         :         .         .         .] 81
mir827     100.0%  TGTGTTTGTTGATTGACATCTATGCATATTGATCATCCTCGTGTTGATGATCGATTCGTTTAGATGACCATCAACAAATAC
17_12      100.0%  ----TTTGTTGATTGACATCTATGC--------------------------------------------------------
197_136    100.0%  -----------------------------------------------------------TTAGATGACCATCAACAAATA-
```

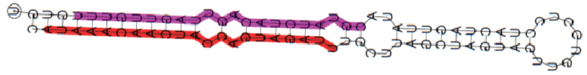

Bna-nMIR394

```
                                1 [         .         .         .         .         :         .         .         .         .         1         .         .         .         .     : ] 152
1 mir394_contig45638 100.0%    CCTTATTGTGTACCTCTCCTGATTACATACGACGGAAAGGAAAGAGAAGAAAGTTTTGATCATGCCGGTTTGACAAAAGAGTTTCTTATAGATATCTTTGGCATTCTGTCCACCTCCTCTACATATGTGTATGGACGATGTGTTCTGTGAGT
2 155_133            100.0%    ------------------------------------------------------------------------------------------------TTTGGCATTCTGTCCACCTCC-----------------------------------
3 506_168            100.0%    -------------------------------------------------------------------------------------------------TTGGCATTCTGTCCACCTCC-----------------------------------
```

Bna-nMIR396

```
                                1 [         .         .         .         .         :         .         .         .         .         ] 100
1 mir396_contig77341 100.0%    AAATAACTAAAAATCTCTAACATTTAACACTCTAGAAAAAAAAAGCTCAAGAAAGCTGTGGGAAAACATGACAATTCAGGGTTTTCTTCATTGATTCAAG
2 10_277             100.0%    --------------------------------------------GCTCAAGAAAGCTGTGGGAAA-----------------------------------
3 0_19               100.0%    -------------------------------------------AGCTCAAGAAAGCTGTGGGAA------------------------------------
4 12_199             100.0%    ---------------------------------------------CTCAAGAAAGCTGTGGGAAA-----------------------------------
5 7_169              100.0%    ------------------------------------------AAGCTCAAGAAAGCTGTGGGA-------------------------------------
6 0_14               100.0%    ---------------------------------------------CTCAAGAAAGCTGTGGGAAAA----------------------------------
7 0_8                100.0%    --------------------------------------------GCTCAAGAAAGCTGTGGGA-------------------------------------
8 0_11               100.0%    ---------------------------------------------------AAAGCTGTGGGAAAACATGAC----------------------------
9 0_10               100.0%    ----------------------------------------------------AAGCTGTGGGAAAACATGACA---------------------------
```

Bna-nMIR01

```
                        1 [         .         .         .         .         :         .         .         .         .         1         .         .         ] 129
mir01-1      100.0%   TCTTTTTGTCTGGGTGATAACAAGATAAATCCCAAGCATCATCCACCTTATTAGCTTTGACATAAACGAAGGCTTGTAAAATGGAAGATTCTTGGGATTTATCTTTTTGTAGTCTCCTCTTCTAAAAGA
2400_1010    100.0%   ------------------------ATAAATCCCAAGCATCATCCA------------------------------------------------------------------------------------
52_10        100.0%   -------------------------TAAATCCCAAGCATCATCCA------------------------------------------------------------------------------------
19_1         100.0%   ------------------------ATAAATCCCAAGCATCATCC-------------------------------------------------------------------------------------
7_0          100.0%   ---------------------AAGATAAATCCCAAGCATCATCCA------------------------------------------------------------------------------------
115_23       100.0%   -----------------------------------------------------------------------------------GAAGATTCTTGGGATTTATCT-------------------------
```

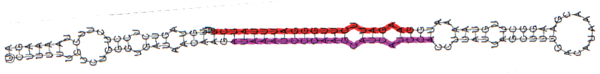

```
                        1 [         .         .         .         .         :         .         .         .         .         1         .         .         .         ] 137
mir01-2      100.0%   CGTTCACTCTTTTGTCAGGTATTAACAAGATAAATCCCAAGCATCATCCACCTTATTAGTCCTTGAACATGAAGGCTTGTAAAATGGATTGATGCTTGGGATTTATTTTGTACTCTCCTCTTCTAAAAGAGTGTTGC
2400_1010    100.0%   -----------------------------ATAAATCCCAAGCATCATCCA---------------------------------------------------------------------------------------
52_10        100.0%   ------------------------------TAAATCCCAAGCATCATCCA---------------------------------------------------------------------------------------
19_1         100.0%   -----------------------------ATAAATCCCAAGCATCATCC----------------------------------------------------------------------------------------
7_0          100.0%   --------------------------AAGATAAATCCCAAGCATCATCCA---------------------------------------------------------------------------------------
4_0          100.0%   --------------------------------------------------------------------------------------GATTGATGCTTGGGATTTATT------------------------------
2_1          100.0%   -------------------------------------------------------------------------------------------ATGCTTGGGATTTATTTTGTA-------------------------
```

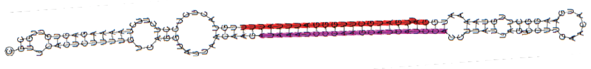

Bna-nMIR02

```
                      1 [      .      .      .      .      :      .      .      .      .      1      .  ] 114
mir02      100.0%   TTGAGCACATATTTATCTTGGTTTTGAGATCTTCTACATGGTATCAAAGCCATGTTATCATTGATAGCCTGGCTCTGATACCATGAAGTAAATCTTAAAACAAGACTTGCTCAG
856_286    100.0%   ----------------------TTTGAGATCTTCTACATGGTA-----------------------------------------------------------------------
5_0        100.0%   ------------------------TGAGATCTTCTACATGGTATC---------------------------------------------------------------------
429_56     100.0%   -----------------------------------------------------------------AGCCTGGCTCTGATACCATGAAGT-------------------------
77_4       100.0%   ---------------------------------------------------------------------TGGCTCTGATACCATGAAGTA------------------------
24_0       100.0%   -----------------------------------------------------------------AGCCTGGCTCTGATACCATGAAG--------------------------
15_3       100.0%   --------------------------------------------------------------------CTGGCTCTGATACCATGAAGT-------------------------
14_1       100.0%   ------------------------------------------------------------------GCCTGGCTCTGATACCATGAAGTA------------------------
9_3        100.0%   -----------------------------------------------------------------AGCCTGGCTCTGATACCATGAAGTA------------------------
6_0        100.0%   -------------------------------------------------------------------------TCTGATACCATGAAGTAAATC--------------------
```

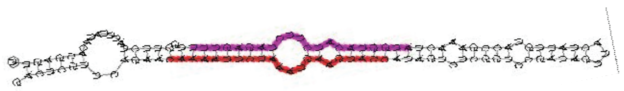

Bna-nMIR03

```
                      1 [      .      .      .      .      :      .      .      .      .      1      .  ] 113
mir03      100.0%   GGATGGTTTCAGGGATTAAGTGTCTGTATGTATGGGCTGAGTAGGCACAAGATGCTGGATTTGTGATTGTACCTTCTCCACCCATACCATACAGACCCTTAATCCTGAATCCA
114_29     100.0%   ---------------------GTCTGTATGTATGGGCTGAGT-----------------------------------------------------------------------
119_13     100.0%   ----------------------TCTGTATGTATGGGCTGAGTA----------------------------------------------------------------------
11_0       100.0%   ---------------------GTCTGTATGTATGGGCTGAGTA----------------------------------------------------------------------
10_3       100.0%   ---------------TTAAGTGTCTGTATGTATGGG-----------------------------------------------------------------------------
10_0       100.0%   ---------------------GTCTGTATGTATGGGCTGAGTAGG--------------------------------------------------------------------
7_3        100.0%   ----------------------TCTGTATGTATGGGCTGAGT-----------------------------------------------------------------------
5_1        100.0%   -------------------GTGTCTGTATGTATGGGCT---------------------------------------------------------------------------
504_155    100.0%   ---------------------------------------------------------------------------TCCACCCATACCATACAGACCC----------------
9_0        100.0%   -----------------------------------------------------------------------------CCACCCATACCATACAGACCCT--------------
7_2        100.0%   ---------------------------------------------------------------------------TCCACCCATACCATACAGACC-----------------
7_0        100.0%   ---------------------------------------------------------------------------TCCACCCATACCATACAGA-------------------
```

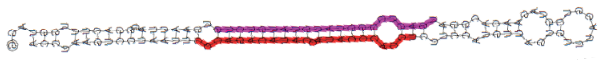

Bna-nMIR04

Sequence	Identity	Aligned sequence (positions 1–183)
mir04	100.0%	TCCTGTACTGATGAGTTCACACTGATTTTGCGTTTCAACTCGGTCCTCATTTCTTTATAACGAAGTGGAGGAACAAAAAGATGTTTTCAGAAGAACATTGAAGAGGAAGAGAATGAAATCTGCAAGTTAAAAGAAATGAGGGCTGAATTGGAACGCAAAATCTTTGAACTAAAGAGACAAGAT
49_24	100.0%	TTTTGCGTTTCAACTCGGTCC
48_9	100.0%	TGAGGGCTGAATTGGAACGCA
21_27	100.0%	ATGAGGGCTGAATTGGAACGC
29_10	100.0%	GCTGAATTGGAACGCAAAATC
7_1	100.0%	TGAGGGCTGAATTGGAACGCAA
5_5	100.0%	AATGAGGGCTGAATTGGAACGC

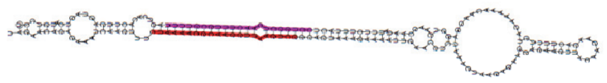

Bna-nMIR05

Sequence	Identity	Aligned sequence (positions 1–441)
mir05	100.0%	GAGAAATCAATAAGTATCCCTATAGTTCTGATTGTCTTAAAATGTGTTAGGACCTAGGAATTCTGAAGTGGCTGGTGGTTACTTTTTCCTTTTGCAGGTGATTGAAGTAGTGGTAAGTATTAGATTAAGTCCTCTTGACACCGCATGTTCGTACCAAGCTAGTTAGAAATTACTAGTGTTGATTGCTTTTGAGTTGTTGTG TGCTAACGCGTTTATCTTGATAACCAGCCTAAGTTGCTAGCTTATCTATTAAAGTAAGTGTGTTTTGTCATACTGCTGTTGTGTGCTTGATCGCTAATATGGCAGTGCAAAATAGATCCCATTACCTTATGTTCGATGTTGGTTTTGCTTATTAATATGTAATATGACCTGTCAAGCCACAACTTCAGAGTTCCTTTGTCGTCTAAGACGTATCATTAGAACTATAGAGGTACTTGTTGGTT
97_24	100.0%	TAAGTATCCCTATAGTTCTGA
11_5	100.0%	AACTATAGAGGTACTTGTTGG
10_1	100.0%	TAGAACTATAGAGGTACTTGT

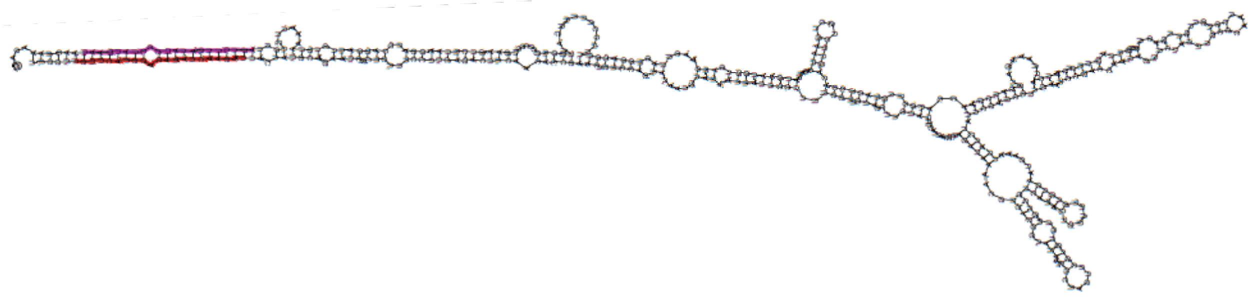

Bna-nMIR06

```
                 1 [        .        .        .        .        :        .        .        .        .        1        .        .        .        .        :        .  ] 163
mir06    100.0%  TTTAGTTAACATCTTCAGAGATCGTCTGATACTAATGGGTCATTTTTTGTAATTGGTAAATCTTAACTAAACATTTTACACGCTTGAGAGACTCTTTCTCTTGCAAAAAATGATCCATTAGTATCAGACGATCTCAAAGACGGTGTGTCCCTCTTAACTAAAC
103_19   100.0%  ---------------------TCGTCTGATACTAATGGGTCA-------------------------------------------------------------------------------------------------------------------------
84_28    100.0%  ------------------AGATCGTCTGATACTAATGGG----------------------------------------------------------------------------------------------------------------------------
9_0      100.0%  -------------------GATCGTCTGATACTAATGGG----------------------------------------------------------------------------------------------------------------------------
72_4     100.0%  -----------------------------------------------------------------------------------------------------------------TCCATTAGTATCAGACGATCT-----------------------------
8_25     100.0%  ----------------------------------------------------------------------------------------------------------------ATCCATTAGTATCAGACGATC------------------------------
13_10    100.0%  --------------------------------------------------------------------------------------------------------------TGATCCATTAGTATCAGACGA--------------------------------
10_1     100.0%  -------------------------------------------------------------------------------------------------------------------CATTAGTATCAGACGATCTCA---------------------------
```

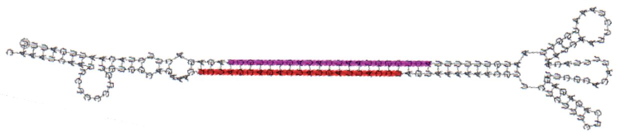

Bna-nMIR07

```
                    1 [        .         .         .         .         :         .         .         .         .         1         .         .         .       ] 137
mir07-1  100.0%   CATGCCGCTCACAGCATCTGAACTCTCTTCATCGTTCAACCAATCATCTGCGTCTTCTTCTTCTTCTACATCCTCAGCTTGCACGTTGACAAATCTAGATGAAGAAGAAGAGAGTTCAGATGCTGTGAGCGACAAAT
7_2      100.0%   -----CGCTCACAGCATCTGAACTCT--------------------------------------------------------------------------------------------------------------
6_2      100.0%   ------------------------------------------------------------------------------------------------------------AGAGAGTTCAGATGCTGTGAG--------
```

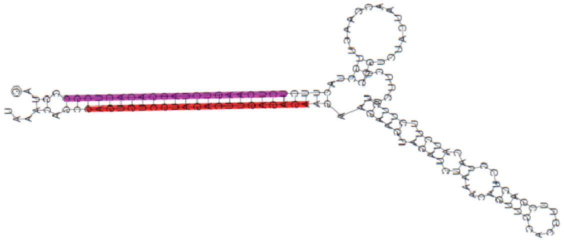

```
                    1 [        .         .         .         .         :         .         .         .         .         1         .         .         .       ] 137
mir07-2  100.0%   ATTTGTCGCTCACAGCATCTGAACTCTCTTCTTCTTCATCTAGATTTGTCAACGTGCAAGCTGAGGATGTAGAAGAAGAAGAAGACGCAGATGATTGGTTGAACGATGAAGAGAGTTCAGATGCTGTGAGCGGCATG
7_2      100.0%   ------CGCTCACAGCATCTGAACTCT-------------------------------------------------------------------------------------------------------------
13_6     100.0%   -----------------------------------------------------------------------------------------------------------------AGTTCAGATGCTGTGAGCGGC---
6_2      100.0%   -------------------------------------------------------------------------------------------------------------AGAGAGTTCAGATGCTGTGAG-------
```

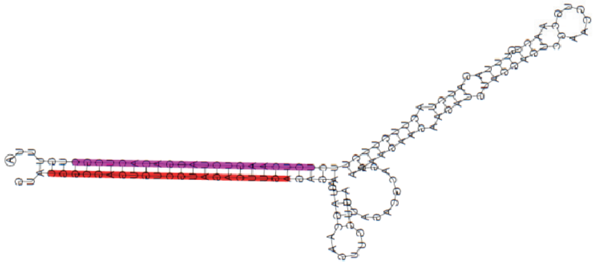

Bna-nMIR08

```
                  1 [         .         .         .         .         :         .         .         .         .         ] 100
mir08   100.0%   TTCAATTTTTATAGTTGTGGCTTTAACATCTTAACTAGAATCATGCTTTGAATGATTAACATGTGAAGTATTGTTGTGTATGTTTTAGCCTACAGTGAAC
3_7     100.0%   ---------------TGTGGCTTTAACATCTTAACTA---------------------------------------------------------------
4_3     100.0%   ----------------GTGGCTTTAACATCTTAACTA---------------------------------------------------------------
2_0     100.0%   ---------------TGTGGCTTTAACATCTTAACTAGA-------------------------------------------------------------
3_0     100.0%   ----------------GTGGCTTTAACATCTTAACTAGA-------------------------------------------------------------
1_0     100.0%   ---------------TGTGGCTTTAACATCTTAACTAG--------------------------------------------------------------
1_1     100.0%   ----------------GTGGCTTTAACATCTTAAC-----------------------------------------------------------------
0_2     100.0%   ----------------GTGGCTTTAACATCTTAA------------------------------------------------------------------
0_1     100.0%   ---------------------------------------------------------------TGAAGTATTGTTGTGTATGTT----------------
```

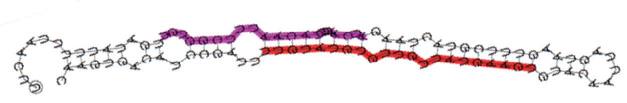

Bna-nMIR09

```
                  1 [         .         .         .         .         :         .         .         .         .         1         .      ] 117
mir09   100.0%   CCTATACATCCCTAATGGTGATTTTGAATCTAAAGTTCTCAAAATACGTATATGTCTTTCCAGTAACTTTAGGTTGAGAATCACCATTGGGGCTCTTTAGAAGCTTCCAACGCTGCA
8_0     100.0%   ------------TAATGGTGATTTTGAATCTAA------------------------------------------------------------------------------------
7_6     100.0%   -------------------------------------------------------------------------TTGAGAATCACCATTGGGGCT-----------------------
```

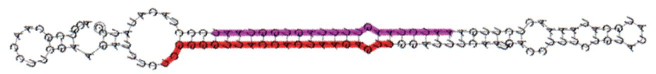

Bna-nMIR11

```
                    1 [         .         .         .         .         :         .         .         .         .         1         ] 110
mir11    100.0%   TATTGACTACTGCGGCAACCAAACGAACATGACTGATGTTGATTCCACATTGCGACGTTATGTGGCATTTTCCTGGGTAAAGAATTAGTGTTTTTTTTTTGTTGCTGCAG
21_14    100.0%   ----------TGCGGCAACCAAACGAACATG-------------------------------------------------------------------------------
4_0      100.0%   --------ACTGCGGCAACCAAACGAACATGA------------------------------------------------------------------------------
```

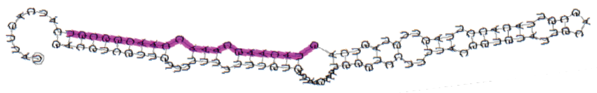

Bna-nMIR12

```
                    1 [         .         .         .         .         :         .         .       ] 77
mir12    100.0%   GATCTCAGTTCGCTTTCTGGTTGATCGCTAATTCGTTTTTAGTTTTTGAATGGCTTCATCAGATAGTCGATGTGGAT
2_8      100.0%   --------------------------------------------------TGGCTTCATCAGATAGTC---------
0_2      100.0%   --------------------------------------------------TGGCTTCATCAGATAGTCGATGT----
0_3      100.0%   --------------------------------------------------TGGCTTCATCAGATAGTCGAT-----
```

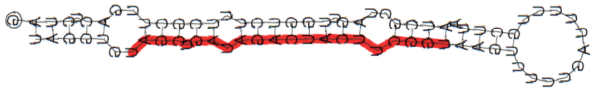

Bna-nMIR13

```
                 1 [        .         .         .         .         :         .        ] 70
mir13   100.0%  GGTGAACCGGGCCTGTTTCTCCTCTTGCGTTGCTAAGCCTGGCGCGAAGCGAAGGGATTGGATTTCACCT
9_0     100.0%  ------------------------------------GCCTGGCGCGAAGCGAAGGGA-------------
2_0     100.0%  -----------------------------------AGCCTGGCGCGAAGCGAAGGG--------------
0_1     100.0%  -----------------------------------AGCCTGGCGCGAAGCGAAGGGA-------------
1_0     100.0%  ------------------------------------GCCTGGCGCGAAGCGAAGGG--------------
```

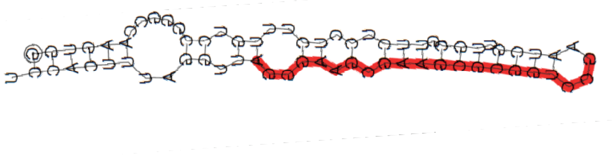

Bna-nMIR14

```
               1 [        .         .         .         .         :         .         .         .         .         1         .         .        . ] 132
mir14   100.0%  CGAAAGAGCGCGACGTCGTCGCTTGTTTCTTCGCCGGAAAGCCATCGTTATCCCAGAGGAAGACACCGTGGCCTCGTCGCACTGATAAAATATGTTTCCCGGCGGAAGAAACAAACGACGCCGACGACTGTT
68_10   100.0%  ------------------TCGCTTGTTTCTTCGCCGGAA---------------------------------------------------------------------------------------------
3_0     100.0%  ------------------TCGCTTGTTTCTTCGCCGGA----------------------------------------------------------------------------------------------
```

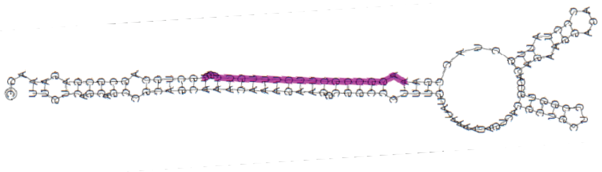

Supplement: Figure S3 — Predicted secondary structures of new pre-miRNAs in B. napus . Secondary structures of candidate miRNA precursors of new B. napus miRNA families (Bna-nMIRx), their locations and the abundance of small RNAs mapped onto these precursors. Mature miRNAs located in the 5p and 3p arms are labeled in magenta and red, respectively. Values on the left side of the miRNA sequence represent read counts in the mature and developing seed libraries, respectively. (PDF) [file pone.0050663.s003.pdf]
